# Comparison between Calcitriol and Calcitriol Plus Low-Dose Cinacalcet for the Treatment of Moderate to Severe Secondary Hyperparathyroidism in Chronic Dialysis Patients

**DOI:** 10.3390/nu5041336

**Published:** 2013-04-19

**Authors:** Yueh-Ting Lee, Hwee-Yeong Ng, Chien-Chun Kuo, Te-Chuan Chen, Chien-Shing Wu, Terry Ting-Yu Chiu, Wen-Chin Lee, Chien-Te Lee

**Affiliations:** Division of Nephrology, Department of Internal Medicine, Kaohsiung Chang Gung Memorial Hospital, Chang Gung University College of Medicine, Kaohsiung, 833, Taiwan; E-Mails: yuai@adm.cgmh.org.tw (Y.-T.L.); kujiben@gmail.com (H.-Y.N.); gary@adm.cgmh.org.tw (C.-C.K.); puppy@adm.cgmh.org.tw (T.-C.C.); vjf3@adm.cgmh.org.tw (C.-S.W.); tytc107@gmail.com (T.T.-Y.C.); leewc@adm.cgmh.org.tw (W.-C.L.)

**Keywords:** cinacalcet, uremic hyperparathyroidism, dialysis, parathyroid hormone

## Abstract

Aim: Uremic hyperparathyroidism (UHPT) has been shown to contribute to the development and progression of chronic kidney disease—mineral bone disorder. UHPT is frequently observed in chronic dialysis patients, and patients with UHPT are associated with increased risk of all-cause and cardiovascular mortality. Cinacalcet is a novel agent that increases sensitivity to the calcium-sensing receptor and is approved for control of UHPT. Nevertheless, cinacalcet is costly and information regarding efficacy of low-dose cinacalcet on UHPT is limited. Methods: We conducted a retrospective study to evaluate treatment with either low-dose calcitriol combined with low-dose cinacalcet (25 mg) (d-Cinacalcet) or calcitriol alone (VitD) in dialysis patients with moderate to severe UHPT. A total of 81 dialysis patients were enrolled (40 subjects in d-Cinacalcet group and 41 subjects in VitD group). Demographic data including age, gender, duration on dialysis and biochemical data were reviewed and recorded. Results: At the end of the study, the intact parathyroid hormone (iPTH) levels of the d-Cinacalcet group declined significantly (from 1166.0 ± 469.3 pg/mL to 679.8 ± 421.6 pg/mL, *p* < 0.0001), while there was no significant change in the VitD group. Significant decrease of serum calcium (Ca: 9.9 ± 0.6 mg/dL *vs.* 9.6 ± 0.8 mg/dL, *p* = 0.002), phosphorus (P: 5.9 ± 1.3 mg/dL *vs.* 4.9 ± 0.9 mg/dL, *p <* 0.0001) and calcium phosphate product (Ca × P: 58.7 ± 15.0 mg^2^/dL^2^* vs.* 46.9 ± 8.9 mg^2^/dL^2^, *p* < 0.0001) were observed in the d-Cinacalcet group. In addition, the subjects in the d-Cinacalcet group had a greater proportion to achieve Kidney Disease Outcomes Quality Initiative (KDOQI)-recommended biochemical targets than the subjects in the VitD group (Ca: 48% *vs.* 24%; P: 78% *vs.* 32%; Ca × P: 85% *vs.* 37%; iPTH: 15% *vs.* 0%). Conclusions: We conclude that combination therapy of low-dose cinacalcet and calcitriol is more effective than calcitriol alone as a treatment for moderate and severe UHPT in chronic dialysis patients. Furthermore, this therapy is associated with improvement in hyperphosphatemia and hypercalcemia.

## 1. Introduction

Uremic hyperparathyroidism (UHPT) is one of the complications of chronic kidney disease (CKD) and is more commonly observed in patients undergoing dialysis. The pathogenesis of UHPT is complex and multiple. The major factors responsible for stimulating parathyroid gland function in renal failure involve hyperphosphatemia, hypocalcemia, decreased production of 1,25-dihydroxyvitamin D and peripheral resistance to parathyroid hormone (PTH) [1,2]. Furthermore, reduced expression of vitamin D receptor (VDR) and calcium-sensing receptor (CaSR), down-regulation of klotho-fibroblast growth factor receptor (FGFR) complex, instability of PTH mRNA of parathyroid gland also play important roles in the development and progression of UHPT [3,4,5]. Of note, if physiologic disturbances of UHPT are not corrected, renal osteodystrophy will develop and lead to weakness, fractures, bone muscle pain, and avascular necrosis. Meanwhile, as described in our previous study, the disturbance of bone mineral metabolism was associated with chronic inflammation in chronic dialysis patients [6]. Recent studies also demonstrated the significant association of CKD—mineral bone disorder and increased risk of all-cause and cardiovascular mortality as a consequence of accelerated atherosclerosis and arterial calcification [7,8,9].

Therefore, prevention and control of UHPT is crucial for dialysis patients as the excessive parathyroid hormone exerts systemic toxicity. During the past decades, treatment of UHPT has been more advanced and evolved based on new insights into its pathogenesis. The major principle is to control UHPT and maintain normal value of blood concentration of calcium and phosphorous. Higher dose pharmacological use of vitamin D therapy through the activation of VDR has demonstrated its efficacy in suppressing hyperparathyroidism and several large observational studies have also shown survival advantage [10,11,12]. Nevertheless, use of high dose vitamin D is associated with exacerbating hyperphosphatemia and causing hypercalcemia, as well as the potential risk of cardiovascular calcification [13]. Calcimimetic agent, such as cinacalcet, which not only allosterically enhances sensitivity to CaSR, but also increases the expression of VDR of the parathyroid gland, became available since its approval in 2004 [14]. In contrast to vitamin D, the calcimimetic agent can significantly lower serum of the intact parathyroid hormone (iPTH) and simultaneously reduce serum calcium and phosphorous levels [15]. In addition, Block *et al.* reported its benefit of cardiovascular protection in a large observational study for hemodialysis subjects [16]. More recently, considerable evidences also exhibited that combination administration of a low dose of vitamin D and stepped-up cinacalcet provide apparent efficacy in treating UHPT [17,18,19]. 

However, despite the recommended target goals that have been clarified by the Kidney Disease Outcomes Quality Initiative (K/DOQI) [20] and Kidney Disease: Improving Global Outcomes (KDIGO) [21], the appropriate medical management for moderate to severe UHPT of dialysis patients, especially for those who do not expect or refuse a parathyroidectomy, has not been well defined in addition to its already limited information [18,19]. Thus, we conducted a retrospective study to assess efficacy of either calcitriol combined with low-dose cinacalcet (25 mg per day) (d-Cinacalcet) or calcitriol alone (VitD) in dialysis patients with moderate to severe UHPT.

## 2. Experimental Section

### 2.1. Study Population

In the retrospective study, adult uremic patients those have been receiving chronic dialysis therapy for at least 3 months in our hospital were eligible for screening. Clinically, these dialysis patients received either oral or intravenous calcitriol therapy for UHPT when the iPTH level was greater than 300 pg/mL. The candidate characteristics of subjects include moderate to severe UHPT with the iPTH level above 500 pg/mL and under treatment with either calcitriol combined with low-dose cinacalcet (Regpara 25 mg per day) (d-Cinacalcet) or calcitriol alone (VitD) continuously for at least 6 months. Patients with a previous parathyroidectomy were excluded. Cinacalcet was maintained at 25 mg until iPTH was less than 150 pg/mL. The dosage of the phosphate binder and vitamin D sterols, either oral or intravenous calcitriol, was elastically titrated or discontinued temporarily according to iPTH and calcium phosphate product (Ca × P) levels as per K/DOQI and KDIGO guidelines recommendation. Eight hundred and seventy-three subjects between January 2011 and June 2012 attending our dialysis unit were reviewed, and a total of 81 patients (40 subjects in the d-Cinacalcet group containing 23 hemodialysis and 17 peritoneal dialysis, 41 subjects in VitD group containing 37 hemodialysis and 4 peritoneal dialysis) were eligible for further analysis. All patients were dialyzed with either 4 h of hemodialysis per session and 3 sessions per week via arteriovenous fistula or graft using bicarbonate-based dialysate containing calcium 2.5 mEq/L or 4–5 exchanges per day of continuous ambulatory peritoneal dialysis with glucose-based solutions (Dianeal solution, Baxter,Singapore) containing calcium 2.5 mEq/L. 

### 2.2. Demographic and Laboratory Data

The detailed demographic data, including age, gender, dialysis duration, modality of renal replacement therapy, adequacy of dialysis (using Kt*/*V by kinetic model of Daugirdas for hemodialysis subjects and using peritoneal weekly Kt/V for peritoneal dialysis subjects), underlying renal disease of end-stage renal disease, and comorbidity (diabetes and hypertension) were reviewed. The dosage of phosphate binder and vitamin D sterols and biochemical data such as corrected serum calcium, phosphorous, iPTH, alkaline phosphatase and hemoglobin were collected and recorded monthly for a successive 6 months before and after commencing UHPT therapy. The baseline data of biochemical parameters represent the averaged values of 6 months prior to the initiation of treatment. 

The longitudinal reduction rate of iPTH from baseline and the proportion of patients reaching K/DOQI (corrected serum Calcium 8.4–9.5 mg/dL, phosphrous <5.5 mg/dL, Ca × P < 55 mg^2^/dL^2^, iPTH < 300 pg/mL) and KDIGO (two to nine times the upper normal limit for the assay of iPTH) guidelines at the end of the study were calculated individually. The iPTH was determined using direct chemiluminescent immunoassay (Intact PTH; Siemens Healthcare Diagnostics, INC, Erlangen, Germany) with a normal range of 12–72 pg/mL. Serum calcium and phosphorous levels were analyzed by an onsite laboratory using standard methods. Serum albumin levels were determined by the bromocresol green method and corrected serum calcium levels were accessed as follows: corrected serum calcium (mg/dL) = measured serum calcium (mg/dL) + 0.8 × (4.0 − serum albumin (mg/dL)). 

### 2.3. Statistical Analysis

Statistical analysis was performed using SPSS version 17.0 (SPSS Inc., Chicago, IL). All categorical data were provided for absolute counts or percentages, whereas mean value ± standard deviation (SD) was presented as continuous data. To compare baseline and longitudinal differences in diverse treatment groups, Fisher exact test and unpaired *t* test were used for categorical data and continuous variables analysis, respectively. Analysis of longitudinal within-group continuous data was assessed by paired tests. A *p* value <0.05 was attributed as statistically significant.

### 2.4. Ethical Issues

The study was approved by the Institutional Review Board of Chang-Gung Memorial Hospital (IRB number: 101-4851B). 

## 3. Results

### 3.1. Baseline Characteristics between Different Treatment Groups (Table 1)

Table 1 shows the baseline demographic and laboratory parameters of enrolled patients. Among 81 subjects, approximately three-quarters had severe UHPT (defined as having an iPTH level greater than 800 pg/mL) and one-quarter of the patients had moderate UHPT (defined as iPTH between 500 and 800 pg/mL). The mean age, gender, dialysis duration, primary cause of renal failure and proportion of diabetes and hypertension are similar between the two groups. The distribution of UHPT severity did not differ between the two groups. There were no significant differences in their baseline levels of iPTH, calcium, phosphorous, calcium phosphate product (Ca × P), alkaline phosphatase and hemoglobin. In addition, the parameter of adequacy of dialysis was similar between d-Cinacalcet (hemodialysis: Kt/V: 1.47 ± 0.38; peritoneal dialysis: weekly Kt/V: 1.82 ± 0.34) and VitD groups (hemodialysis: Kt/V: 1.40 ± 0.32; peritoneal dialysis: weekly Kt/V: 1.75 ± 0.41).

**Table 1:** Baseline demographic and biochemical data (*N* = 81).

	All (*N* = 81)	d-Cinacalcet (*N* = 40)	VitD (*N* = 41)	*p* value
Age (years)	55.5 ± 12.6	54.1 ± 11.3	57.0 ± 13.8	0.304
Gender (%)				0.750
Male	33 (41)	17 (43)	16 (39)	
Female	48 (59)	23 (57)	25 (61)	
Dialysis duration (months)	99.4 ± 51.5	89.4 ± 52.3	109.2 ± 49.5	0.085
Primary cause of renal failure (%)				0.293
Chronic glomerulonephritis	49 (60)	27 (68)	22 (54)	
Diabetes mellitus	9 (11)	3 (7)	6 (15)	
Polycystic kidney disease	2 (3)	2 (5)	0 (0)	
Hypertension	12 (15)	4 (10)	8 (19)	
Obstructive nephropathy	9 (11)	4 (10)	5 (12)	
Comorbidity (%)				
Diabetes mellitus	12 (15)	4 (10)	8 (20)	0.228
Hypertension	47 (58)	22 (55)	25 (61)	0.586
Severity of UHPT * (%)				0.396
Moderate (iPTH: 500–800 pg/mL)	19 (23)	11 (27.5)	8 (19.5)	
Severe (iPTH > 800 pg/mL)	62 (77)	29 (72.5)	33 (80.5)	
Calcium (mg/dL)	9.8 ± 0.6	9.9 ± 0.6	9.7 ± 0.6	0.065
Phosphorus (mg/dL)	5.8 ± 1.1	5.9 ± 1.3	5.7 ± 0.8	0.373
Ca × P (mg^2^/dL^2^)	56.7 ± 12.2	58.7 ± 15.0	54.8 ± 8.4	0.157
intact PTH (pg/mL)	1140.4 ± 450.7	1166.0 ± 469.3	1115.5 ± 436.2	0.618
Albumin (mg/dL)	3.9 ± 0.3	3.9 ± 0.2	3.9 ± 0.4	0.319
Alkaline phosphate (U/L)	139.2 ± 90.4	145.4 ± 87.6	133.2 ± 93.8	0.548
Hemoglobin (mg/dL)	10.4 ± 1.3	10.4 ± 1.4	10.4 ± 1.1	0.966
nPNA (g/kg/day) ^┼^	1.15 ± 0.42	1.16 ± 0.38	1.14 ± 0.49	0.586

* uremic hyperparathyroidism: UHPT; ^┼^ total nitrogen appearance normalized to body weight: nPNA.

### 3.2. Comparison of Longitudinal Evolution of CKD-Mineral Bone Disease Parameters Stratified by Diverse Treatment Modalities (Table 2)

The longitudinal changes of parameters of CKD-mineral bone disease at baseline, 3 months, and 6 months after initiating treatment of moderate to severe UHPT is displayed in Table 2. Notably, patients in the d-Cinacalcet group had lower levels of serum calcium, phosphorous, Ca × P, and iPTH and there was a greater reduction rate of iPTH than that in the VitD group at the same time points. No significant difference was noted in levels of alkaline phosphatase, hemoglobin, and albumin between the two groups. With-in group comparison at the end of the study reveals iPTH levels of d-Cinacalcet group declined significantly from 1166.0 ± 469.3 pg/mL to 679.8 ± 421.6 pg/mL (*p* < 0.0001) while there was no significant change in the VitD group. Meanwhile, subjects in the d-Cinacalcet group had a significant decrease in levels of serum calcium (9.9 ± 0.6 mg/dL *vs.* 9.6 ± 0.8 mg/dL, *p* = 0.002; reduction rate: 3.5%), phosphorus (5.9 ± 1.3 mg/dL *vs.* 4.9 ± 0.9 mg/dL, *p<*0.0001; reduction rate: 14.3%) and Ca × P (58.7 ± 15.0 mg^2^/dL^2^* vs.* 46.9 ± 8.9 mg^2^/dL^2^, *p* <0.0001; reduction rate: 17.0%), but subjects in the VitD group had an elevation of serum calcium (9.7 ± 0.6 mg/dL *vs.* 10.0 ± 0.6 mg/dL, *p =* 0.002). 

**Table 2:** Serial changes of 81 patients in biochemical parameters.

	d-Cinacalcet (*N* = 40)	VitD (*N* = 41)	*p* value
**Calcium (mg/dL)**			
Baseline	9.9 ± 0.6	9.7 ± 0.6	0.065
3 months	9.4 ± 0.7 ^a^	9.8 ± 0.6	0.004
Reduction rate (%)	5.6 ± 6.9	−1.2 ± 6.0	<0.0001
6 months	9.6 ± 0.8 ^b^	10.0 ± 0.6 ^b^	0.018
Reduction rate (%)	3.5 ± 7.1	−2.9 ± 5.1	<0.0001
**Phosphorus (mg/dL)**			
Baseline	5.9 ± 1.3	5.7 ± 0.8	0.373
3 months	5.4 ± 1.2 ^a^	6.0 ± 1.0	0.032
Reduction rate (%)	5.8 ± 16.6	−6.4 ± 18.4	0.003
6 months	4.9 ± 0.9 ^bc^	5.7 ± 0.9	<0.0001
Reduction rate (%)	14.3 ± 17.3	−2.9 ± 20.5	<0.0001
**Ca** **×** **P (mg^2^/dL^2^)**			
Baseline	58.7 ± 15.0	54.8 ± 8.4	0.157
3 months	51.0 ± 11.3 ^a^	58.4 ± 10.2 ^a^	0.003
Reduction rate (%)	10.5 ± 19.8	−7.7 ± 20.2	<0.0001
6 months	46.9 ± 8.9 ^bc^	56.9 ± 9.0	<0.0001
Reduction rate (%)	17.0 ± 20.0	−5.7 ± 20.8	<0.0001
**intact PTH (pg/mL)**			
Baseline	1166.0 ± 469.3	1115.5 ± 436.2	0.618
3 months	838.0 ± 418.4 ^a^	1152.7 ± 728.4	0.024
Reduction rate (%)	27.3 ± 21.1	3.7 ± 46.4	0.005
6 months	679.8 ± 421.6 ^bc^	1021.9 ± 655.1	0.007
Reduction rate (%)	40.1 ± 26.5	7.7 ± 40.8	<0.0001
**Albumin (mg/dL)**			
Baseline	3.9 ± 0.2	3.9 ± 0.4	0.319
3 months	3.8 ± 0.4	3.9 ± 0.2	0.104
6 months	3.7 ± 0.4	3.7 ± 0.3	0.503
**Alkaline phosphate (U/L)**			
Baseline	145.4 ± 87.6	133.2 ± 93.8	0.548
3 months	139.7 ± 76.3	130.0 ± 92.0	0.609
6 months	132.9 ± 90.6	130.2 ± 102.9	0.900
**Hemoglobin (mg/dL)**			
Baseline	10.4 ± 1.4	10.4 ± 1.1	0.966
3 months	10.8 ± 1.3	10.3 ± 1.1	0.109
6 months	10.6 ± 1.2	10.4 ± 1.2	0.337

^a^ Comparison between baseline and 3 months, *p* < 0.05; ^b^ Comparison between baseline and 6 months, *p* < 0.05; ^c^ Comparison between 3 months and 6 months, *p* < 0.05.

### 3.3. Goal Attainment of K/DOQI and KDIGO

**Figure 1:**
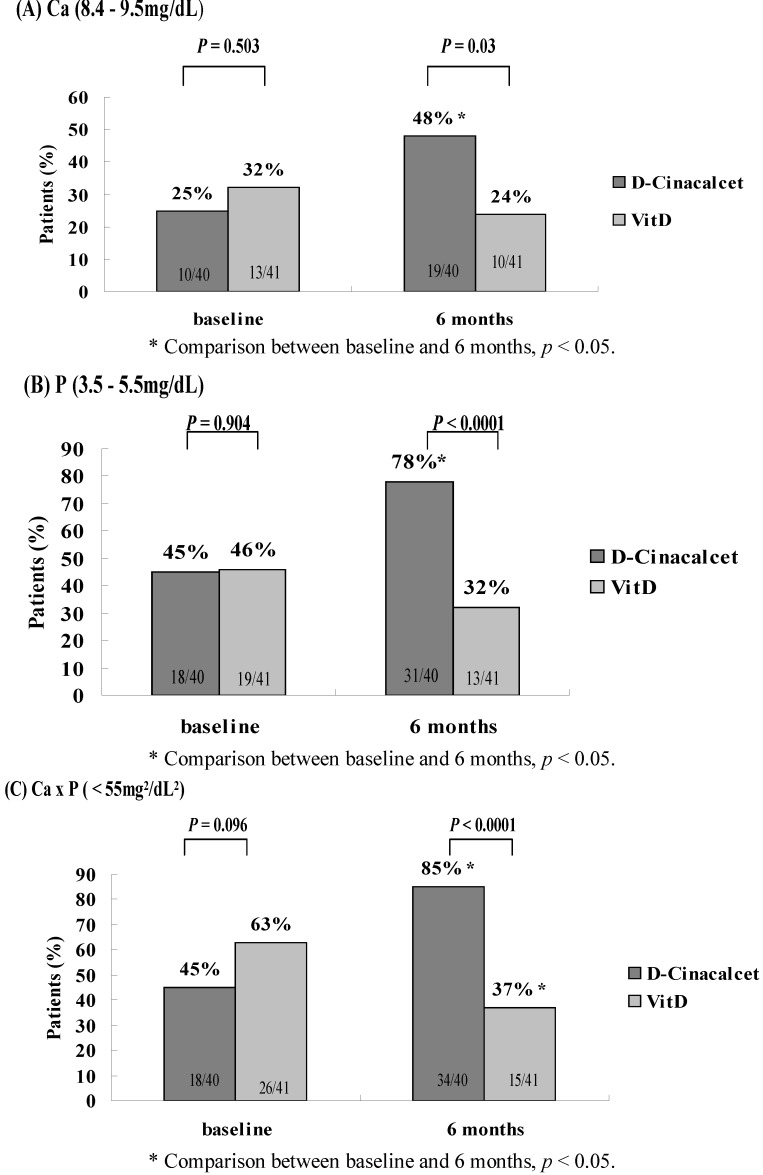
Percentages of patients who achieved the biochemical targets including (**A**) Ca: 8.4–9.5 mg/dL; (**B**) P: 3.5–5.5 mg/dL; and (**C**) Ca × P < 55 mg^2^/dL^2^, recommended by the Kidney Disease Outcomes Quality Initiative (K/DOQI) at baseline and after treatment of 6 months.

**Figure 2:**
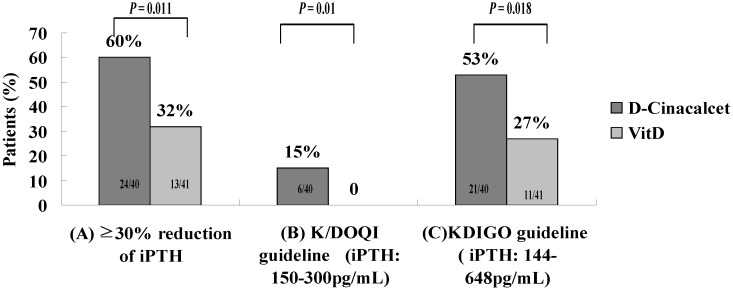
Proportion of patients with (**A**) ≥30% reduction of iPTH from baseline, (**B**) achieving treatment target of iPTH recommended by the Kidney Disease Outcomes Quality Initiative (K/DOQI) and, (**C**) by Kidney Disease: Improving Global Outcomes (KDIGO) after 6 months of treatment.

Figure 1, Figure 2 illustrate the numbers and frequency of subjects achieving the K/DOQI and KDIGO guidelines after a 6-month treatment. At baseline, there was no significant difference in any biochemical target. At the end of the study, the subjects in the d-Cinacalcet group had greater proportion to achieve K/DOQI-recommended targets than the subjects in VitD group (Ca: 48% *vs.* 24%; P: 78% *vs.* 32%; Ca × P: 85% *vs.* 37%; iPTH: 15% *vs.* 0%). In addition, our study revealed that proportion of reduction rate of iPTH levels more than 30% and the rate of achieving KDIGO goals for iPTH levels at 6-month management was significantly higher in subjects in the d-Cinacalcet group over the VitD group (60% *vs.* 32%, *p =* 0.011; 53% *vs.* 27%, *p =* 0.018). Three patients (7.5%) in the d-Cinacalcet group and 1 patient (2.4%) in the VitD group developed hypocalcemia (*p =* 0.293). None of the subjects in either group received native vitamin D or parathyroidectomy during the study period. 

### 3.4. The Use and Dosage of Phosphate Binder and Calcitriol

No significant difference was noted in the prescription pattern and dosage of calcitriol during a 6-month period before commencing treatment of UHPT among d-Cinacalcet group (intravenous calcitriol (*N* = 25): 1.21 ± 0.83 μg/week; oral calcitriol (*N* = 15): 2.13 ± 0.76 μg/week) and VitD group (intravenous calcitriol (*N* = 23): 1.17 ± 0.77 μg/week; oral calcitriol (*N* = 18): 2.21 ± 0.82 μg/week). The usage of of phosphate binder and calcitriol during treatment period is summarized in Table 3. At the beginning of therapy, subjects in the VitD group had a greater proportion of administration of phosphate binder to control hyperphosphatemia. At the end of study, decrease in calcitriol usage and mean weekly dosage of intravenous calcitriol was observed in the VitD group. Nevertheless, for subjects in the d-Cinacalcet group, we found the most remarkable change is a decrease in administration and mean daily dosage of aluminum hydroxide use (60% *vs.* 30%, *p =* 0.002; 1333 ± 481 mg/day *vs.* 1166 ± 389 mg/day, *p =* 0.041) and dosage of calcitriol remained constant. 

**Table 3:** Use and dosage of calcitriol and phosphate binders at baseline and after treatment of 6 months.

	Baseline			6 months		
	d-Cinacalcet (*N* = 40)	VitD (*N* = 41)	*p* value	d-Cinacalcet (*N* = 40)	VitD (*N* = 41)	*p* value
**Use of ** **calcitriol (%) **	26 (65)	41 (100)	<0.0001	30 (75)	25 (61) *	0.176
Intravenous calcitriol (%)	14 (35)	34 (83)	<0.0001	17 (43)	24 (59) *	0.221
Dose (μg/week)	5.43 ± 0.94	4.94 ± 1.35	0.184	4.47 ± 1.33	4.28 ± 1.50 *	0.226
Oral Calcitriol (%)	12 (30)	7 (17)	0.170	13 (33)	1 (2)*	<0.0001
Dose (μg/week)	2.17 ± 0.58	2.05 ± 0.21	0.124	2.27 ± 0.88	2.31 ± 0.35	0.773
**Use of phosph** **ate binders (%)**	33 (83)	40 (98)	0.023	28 (70)	37 (90)	0.022
Aluminum hydroxide (%)	24 (60)	36 (88)	0.004	12 (30) *	31 (76)	<0.0001
Dose (mg/day)	1333 ± 481	1167 ± 378	0.161	1166 ± 389 *	1129 ± 341	0.772
Calcium carbonate (%)	11 (28)	15 (37)	0.381	19 (48) *	18 (44)	0.745
Dose (mg/day)	1948 ± 727	1800 ± 621	0.592	1816 ± 628	1705.0 ± 443	0.542
Calcium acetate (%)	4 (10)	6 (15)	0.526	7 (18)	5 (12)	0.502
Dose (mg/day)	2143 ± 861	2293 ± 885	0.806	1920 ± 334	2742 ± 939	0.070

* Comparison between baseline and 6 months, *p* < 0.05.

## 4. Discussion

Our study demonstrated that the combination therapy of low-dose cinacalcet (25 mg per day) and titrated calcitriol is more effective than calcitriol alone as a treatment for moderate to severe UHPT in chronic dialysis patients. This combination therapy is associated with improvement in hyperphosphatemia and hypercalcemia. Furthermore, there was a significant decrease in the usage and dosage of aluminum hydroxide in combination therapy at the end of the treatment period. 

### 4.1. Combination Therapy of Low-Dose Cinacalcet and Calcitriol: A Solution for Moderate to Severe UHPT

For early-stage UHPT, it has been recognized that conventional management with dietary phosphorus restriction, phosphate binder and active vitamin D has distinct efficacy on renal osteodystrophy and additional important health benefits [10,20]. Nevertheless, for severe UHPT, previous studies highlighted that active vitamin D analogs, especially non-selective VDR activator calcitriol, had the potential to raise serum calcium and phosphorus levels, which are accompanied with increased risk for cardiovascular disease mediated through probable vascular calcification by enhancing gastrointestinal absorption of calcium and phosphate [7,8,13,22], thereby limiting its efficacy due to frequent interruption of its usage. We monitored calcium and phosphorous levels every two weeks, and calcitriol was discontinued or the dosage reduced according to guideline recommendation. It is not surprising that only 61% subjects in the VitD group received calcitriol treatment at the end of 6 months. The intermittent interruption and decreased dosage of calcitriol during the treatment period contributed to only modest reduction in iPTH levels with a lesser proportion able to achieve the K/DOQI-recommended biochemical targets in the VitD group. Our study disclosed the reality in clinical practice for calcitriol treatment of moderate to severe UHPT. On the other hand, although a few analyses demonstrated that selective VDR activator paricalcitol reduced the potential of vitamin D toxicity and had a lower risk of hypercalcemia in dialysis patients compared with non-selective VDR activator calcitriol [19,23,24], further analysis on patients with moderate to severe UHPT is mandatory.

Cinacalcet, currently the only available calcimimetic agent, has been proven to make a pronounced dose-dependent decline in the plasma iPTH concentration and a reduction in the levels of calcium and phosphorus via enhancing the sensitivity of CaSR in the parathyroid gland to calcium [15,25,26]. Frazao *et al.* demonstrated cinacalcet had efficacy for UHPT regardless of its severity and a trend toward greater mean absolute reduction in iPTH and phosphorus levels for subjects with higher baseline iPTH levels, particularly in the ≥1000 pg/mL subgroup [22]. Because of diverse mechanisms in the pathogenesis of UHPT, the concept of combination therapy of cinacalcet and low-dose vitamin D emerged, and clinicians can now use vitamin D more actively due to lower calcium and phosphorus levels during cinacalcet treatment. The effect of combination therapy in management of UHPT has been investigated in two previous studies [17,18] and both demonstrated combined therapy had higher achievement of the biochemical targets set by K/DOQI and KDIGO guidelines over vitamin D alone. Our study also showed similar benefits of combined therapy. Interestingly, however, the baseline iPTH levels of our subjects are significantly higher and the dose of cinacalcet is lower than those for a Western population in two studies (mean iPTH level is around 600 pg/mL and mean dose of cinacalcet is around 70 mg per day). Meanwhile, a lower dose of cinacalcet, which is sufficiently effective to suppress iPTH levels, has been observed in Japanese patients [27]. It appears that there is a geographic difference for the effective dose of cinacalcet. One of the possible explanations is genetic variation of CaSR, such as polymorphism, for the diverse response of cinacalcet. [28]. However, further clinical research is still needed to demonstrate the efficacy of a low dose of cinacalcet in Asian patients in comparison with Western patients. 

### 4.2. Combination Therapy of Low-Dose Cinacalcet and Calcitriol to Reduce Hypocalcemia Episodes and Use of Aluminum Hydroxide

The most reported side effect of cinacalcet treatment is gastrointestinal disorders, including nausea, vomiting, and diarrhea [29]. This symptom is generally relieved with food and often resolves with continued use. Furthermore, although hypocalcemia is not uncommon during cinacalcet management, the present study identified that incidence of hypocalcemia was comparable between combined therapy and calcitriol alone. Thus, our findings support the evidence for lesser hypocalcemia when combined therapy is introduced because vitamin D can offset the reduction of calcium levels by cinacalcet treatment.

On the other hand, frequent use of aluminum hydroxide is noted in our study, which is because of the severity of UHPT in our subjects and they were prone to suffer hypercalcemia under administration of calcium-based phosphate binders. Although K/DOQI and KDIGO guidelines highlight its restrictive use for the concern of neurological, skeletal, and hematological toxicity of aluminum [20,21], the use of aluminum hydroxide as a phosphate binder is still one of the options because its high cost effectiveness in lowering phosphorous levels. Nevertheless, after half year of combined therapy, our study revealed a significant decrease in using aluminum hydroxide and even in mean daily dosage. It therefore indicates that combined therapy may reduce the use of aluminum hydroxide and lower the risk of aluminum toxicity in dialysis patients. 

### 4.3. Limitations of the Study

First of all, the study subject was relatively simple with a heterogeneous population (including hemodialysis and peritoneal dialysis). Secondary, retrospective study may not include all patients enrolled initially. Patients who cannot tolerate treatment or who withdrew because of any other reasons will be excluded. Lastly, neither serum bone-specific alkaline phosphatase levels nor bone mineral density, or even bone biopsies, were examined. We cannot support the direct effect of combined therapy on bone turnover, which is an important component of mineral and bone metabolism. Therefore, further prospective long-term studies are necessary to confirm the beneficial effects of a combined therapy of low-dose cinacalcet and calcitriol on renal bone pathology.

## 5. Conclusions

Our study provides supportive evidence that combination therapy of low-dose cinacalcet and calcitriol was effective to treat moderate and severe UHPT. Furthermore, our results demonstrated that low-dose cinacalcet as an add-on therapy to standard treatment including calcitriol and phosphate binders improved goal attainment of CKD—mineral bone disease over low-dose calcitriol alone. 

## References

[1] Levin A., Bakris G.L., Molitch M., Smulders M., Tian J., Williams L.A., Andress D.L. (2007). Prevalence of abnormal serum vitamin D, PTH, calcium, and phosphorus in patients with chronic kidney disease: Results of the study to evaluate early kidney disease. Kidney Int..

[2] Pitts T.O., Piraino B.H., Mitro R., Chen T.C., Segre G.V., Greenberg A., Puschett J.B. (1988). Hyperparathyroidism and 1,25-dihydroxyvitamin D deficiency in mild, moderate, and severe renal failure. J. Clin. Endocrinol. Metab..

[3] Li Y.C., Amling M., Pirro A.E., Priemel M., Meuse J., Baron R., Delling G., Demay M.B. (1998). Normalization of mineral ion homeostasis by dietary means prevents hyperparathyroidism, rickets, and osteomalacia, but not alopecia in vitamin D receptor-ablated mice. Endocrinology.

[4] Panda D.K., Miao D., Bolivar I., Li J., Huo R., Hendy G.N., Goltzman D. (2004). Inactivation of the 25-hydroxyvitamin D 1alpha-hydroxylase and vitamin D receptor demonstrates independent and interdependent effects of calcium and vitamin D on skeletal and mineral homeostasis. J. Biol. Chem..

[5] Krajisnik T., Björklund P., Marsell R., Ljunggren O., Akerström G., Jonsson K.B., Westin G., Larsson T.E. (2007). Fibroblast growth factor-23 regulates parathyroid hormone and 1alpha-hydroxylase expression in cultured bovine parathyroid cells. J. Endocrinol..

[6] Lee C.T., Tsai Y.C., Ng H.Y., Su Y., Lee W.C., Lee L.C., Chiou T.T., Liao S.C., Hsu K.T. (2009). Association between C-reactive proteinandbiomarkers of bone and mineralmetabolism in chronic hemodialysis patients: A cross-sectional study. J. Ren. Nutr..

[7] Block G.A., Klassen P.S., Lazarus J.M., Ofsthun N., Lowrie E.G., Chertow G.M. (2004). Mineral metabolism, mortality, and morbidity in maintenance hemodialysis. J. Am. Soc. Nephrol..

[8] Palmer S.C., Hayen A., Macaskill P., Pellegrini F., Craig J.C., Elder G.J., Strippoli G.F. (2011). Serum levels of phosphorous, parathyroid hormone, and calcium and risks of death and cardiovascular disease in individiuals with chronic kidney disease: A systematic review and meta-analysis. JAMA.

[9] Kalantar-Zadeh K., Kuwae N., Regidor D.L., Kovesdy C.P., Kilpatrick R.D., Shinaberger C.S., McAllister C.J., Budoff M.J., Salusky I.B., Kopple J.D. (2006). Survival predictability of time varying indicators of bone disease in maintenance hemodialysis patients. Kidney Int..

[10] Teng M., Wolf M., Ofsthun M.N., Lazarus J.M., Hernán M.A., Camargo C.A., Thadhani R. (2005). Activated injectable vitamin D and hemodialysis survival: A historical cohort study. J. Am. Soc. Nephrol..

[11] Teng M., Wolf M., Lowrie E., Ofsthun N., Lazarus J.M., Thadhani R. (2003). Survival of patients undergoing hemodialysis with paricalcitol or calcitriol therapy. N. Engl. J. Med..

[12] Shoji T., Shinohara K., Kimoto E., Emoto M., Tahara H., Koyama H., Inaba M., Fukumoto S., Ishimura E., Miki T. (2004). Lower risk for cardiovascular mortality in oral 1alpha-hydroxy vitamin D3 users in a haemodialysis population. Nephrol. Dial. Transplant..

[13] Palmer S.C., McGregor D.O., Macaskill P., Craig J.C., Elder G.J., Strippoli G.F. (2007). Meta-Analysis: Vitamin D compounds in chronic kidney disease. Ann. Intern. Med..

[14] Goodman W.G., Frazao J.M., Goodkin D.A., Turner S.A., Liu W., Coburn J.W. (2000). A calcimimetic agent lowers plasma parathyroid hormone levels in patients with secondary hyperparathyroidism. Kidney Int..

[15] Block G.A., Martin K.J., de Francisco A.L., Turner S.A., Avram M.M., Suranyi M.G., Hercz G., Cunningham J., Abu-Alfa A.K., Messa P. (2004). Cinacalcet for secondary hyperparathyroidism in patients receiving hemodialysis. N. Engl. J. Med..

[16] Block G.A., Zaun D., Smits G., Persky M., Brillhart S., Nieman K., Liu J., St Peter W.L. (2010). Cinacalcet hydrochloride treatment significantly improved all-cause and cardiovascular survival in a large cohort of hemodialysis patients. Kidney Int..

[17] Fishbane S., Shapiro W.B., Corry D.B., Vicks S.L., Roppolo M., Rappaport K., Ling X., Goodman W.G., Turner S., Charytan C. (2008). Cinacalcet HCL and concurrent low-dose vitamin D improves treatment of secondary hyperparathyroidism in dialysis patients compared with vitamin D alone: The ACHIEVE study results. Clin. Am. Soc. Nephrol..

[18] Block G.A., Zeig S., Sugihara J., Chertow G.M., Chi E.M., Turner S.A., Bushinsky D.A, TARGET Investigators (2008). Combined therapy with cinacalcet and low doses of vitamin D sterols in patients with moderate to severe secondary hyperparathyroidism. Nephrol. Dial. Transplant..

[19] Ketteler M., Martin K.J., Cozzolino M., Goldsmith D., Sharma A., Khan S., Dumas E., Amdahl M., Marx S., Audhya P. (2012). Paricalcitol *versus* cinacalcet plus low-dose vitamin D therapy for the treatment of secondary hyperparathyroidism in patients receiving haemodialysis: Results of the IMPACT SHPT study. Nephrol. Dial. Transplant..

[20] National Kidney Foundation (2003). K/DOQI clinical practice guidelines for bone metabolism and disease in chronic kidney disease. Am. J. Kidney Dis..

[21] Kidney Disease: Improving Global Outcomes (KDIGO) CKD-MBD Work Group (2009). KDIGO clinical practice guidelines for the diagnosis, evaluation, prevention, and treatment of chronic kidney disease-mineral and bone disorder (CKD-MBD). Kidney Int..

[22] Frazão J.M., Messa P., Mellotte G.J., Geiger H., Hagen E.C., Quarles L.D., Kerr P.G., Baños A., Dehmel B., Urena P. (2011). Cinacalcet reduces plasma intact parathyroid hormone, serum phosphorus and calcium levels in patients with secondary hyperparathyroidism irrespective its severity. Clin. Nephrol..

[23] Martin K.J., González E.A., Gellens M., Hamm L.L., Abboud H., Lindberg J. (1998). 19-Nor-1-alpha-25-dihydroxyvitamin D2 (paricalcitol) safely and effectively reduces the levels of intact parathyroid hormone in patients on hemodialysis. J. Am. Soc. Nephrol..

[24] Ross E.A., Tian J., Abboud H., Hippensteel R., Melnick J.Z., Pradhan R.S., Williams L.A., Hamm L.L., Sprague S.M. (2008). Oral paricalcitol for the treatment of secondary hyperparathyroidism on hemodialysis or peritoneal dialysis. Am. J. Nephrol..

[25] Antonsen J.E., Sherrard D.J., Andress D.L. (1998). A calcimimetic agent acutely suppresses parathyroid hormone levels in patients with chronic renal failure. Rapid communication. Kidney Int..

[26] Goodman W.G., Hladik G.A., Turner S.A., Blaisdell P.W., Goodkin D.A., Liu W., Barri Y.M., Cohen R.M., Coburn J.W. (2002). The Calcimimetic agent AMG 073 lowers plasma parathyroid hormone levels in hemodialysis patients with secondary hyperparathyroidism. J. Am. Soc. Nephrol..

[27] Fukagawa M., Yumita S., Akizawa T., Uchida E., Tsukamoto Y., Iwasaki M., Koshikawa S., KRN1493 study group (2008). Cinacalcet (KRN1493) effectively decreases the serum intact PTH level with favourable control of the serum phosphorous and calcium levels in Japanese dialysis patients. Nephrol. Dial. Transplant..

[28] Rothe H.M., Shapiro W.B., Sun W.Y., Chou S.Y. (2005). Calcium-Sensing receptor gene polymorphism Arg990Gly and its possible effect on response to cinacalcet HCl. Pharmacogenet. Genomics.

[29] Li D., Shao L., Zhou H., Jiang W., Zhang W., Xu Y. (2013). The efficacy of cinacalcet combined with conventional therapy on bone and mineral metabolism in dialysis patients with secondary hyperparathyroidism: A meta-analysis. Endocrine.

